# E-Cadherin expression in human tumors: a tissue microarray study on 10,851 tumors

**DOI:** 10.1186/s40364-021-00299-4

**Published:** 2021-06-05

**Authors:** Eike Burandt, Felix Lübbersmeyer, Natalia Gorbokon, Franziska Büscheck, Andreas M. Luebke, Anne Menz, Martina Kluth, Claudia Hube-Magg, Andrea Hinsch, Doris Höflmayer, Sören Weidemann, Christoph Fraune, Katharina Möller, Frank Jacobsen, Patrick Lebok, Till Sebastian Clauditz, Guido Sauter, Ronald Simon, Ria Uhlig, Waldemar Wilczak, Stefan Steurer, Sarah Minner, Rainer Krech, David Dum, Till Krech, Andreas Holger Marx, Christian Bernreuther

**Affiliations:** 1 Institute of Pathology, University Medical Center Hamburg-Eppendorf, Martinistr. 52, 20246 Hamburg, Germany; 2 Institute of Pathology, Clinical Center Osnabrueck, Osnabrueck, Germany; 3 Department of Pathology, Academic Hospital Fuerth, Fuerth, Germany

**Keywords:** E-cadherin, Neoplastic tissue, Renal cell cancer, Lobular breast cancer, TMA, Immunohistochemistry

## Abstract

**Background:**

The E-Cadherin gene (*CDH1, Cadherin 1*), located at 16q22.1 encodes for a calcium-dependent membranous glycoprotein with an important role in cellular adhesion and polarity maintenance.

**Methods:**

To systematically determine E-Cadherin protein expression in normal and cancerous tissues, 14,637 tumor samples from 112 different tumor types and subtypes as well as 608 samples of 76 different normal tissue types were analyzed by immunohistochemistry in a tissue microarray format.

**Results:**

E-Cadherin was strongly expressed in normal epithelial cells of most organs. From 77 tumor entities derived from cell types normally positive for E-Cadherin, 35 (45.5%) retained at least a weak E-Cadherin immunostaining in ≥99% of cases and 61 (79.2%) in ≥90% of cases. Tumors with the highest rates of E-Cadherin loss included Merkel cell carcinoma, anaplastic thyroid carcinoma, lobular carcinoma of the breast, and sarcomatoid and small cell neuroendocrine carcinomas of the urinary bladder. Reduced E-Cadherin expression was linked to higher grade (*p* = 0.0009), triple negative receptor status (*p* = 0.0336), and poor prognosis (*p* = 0.0466) in invasive breast carcinoma of no special type, triple negative receptor status in lobular carcinoma of the breast (*p* = 0.0454), advanced pT stage (*p* = 0.0047) and lymph node metastasis in colorectal cancer (*p* < 0.0001), and was more common in recurrent than in primary prostate cancer (*p* < 0.0001). Of 29 tumor entities derived from E-Cadherin negative normal tissues, a weak to strong E-Cadherin staining could be detected in at least 10% of cases in 15 different tumor entities (51.7%). Tumors with the highest frequency of E-Cadherin upregulation included various subtypes of testicular germ cell tumors and renal cell carcinomas (RCC). E-Cadherin upregulation was more commonly seen in malignant than in benign soft tissue tumors (*p* = 0.0104) and was associated with advanced tumor stage (*p* = 0.0276) and higher grade (*p* = 0.0035) in clear cell RCC, and linked to advanced tumor stage (*p* = 0.0424) and poor prognosis in papillary RCC (*p* ≤ 0.05).

**Conclusion:**

E-Cadherin is consistently expressed in various epithelial cancers. Down-regulation or loss of E-Cadherin expression in cancers arising from E-Cadherin positive tissues as well as E-Cadherin neo-expression in cancers arising from E-Cadherin negative tissues is linked to cancer progression and may reflect tumor dedifferentiation.

**Supplementary Information:**

The online version contains supplementary material available at 10.1186/s40364-021-00299-4.

## Background

The E-Cadherin gene (*CDH1, Cadherin 1*), located at 16q22.1 encodes for a calcium-dependent membrane glycoprotein with an important role in cellular adhesion and polarity maintenance. It consists of 5 cadherin repeats in the extracellular domain, one transmembrane domain, and an intracellular domain that binds p120-catenin (p120-ctn) and beta-catenin. The intracellular domain contains a highly-phosphorylated beta-catenin binding site which is essential for E-Cadherin function. In epithelial cells, E-Cadherin-containing intracellular junctions are often adjacent to actin-containing filaments of the cytoskeleton [1–5]. The pivotal role of E-Cadherin is highlighted by its expression starting at the 2-cell stage of mammalian embryonic development [5, 6]. In adult tissues, E-Cadherin – also called epithelial cadherin - is expressed in virtually all epithelial tissues, where it is constantly regenerated with a 5 to 10 h half-life on the cell surface [4, 5]. Loss of E-Cadherin function or expression plays a relevant role in cancer progression [7]. E-Cadherin downregulation diminishes cellular adhesion in epithelial tissues. As a result, increased cell motility may facilitate invasive growth and metastasis [7]. Heterozygous germline alterations of the *CDH1* gene are associated with hereditary diffuse gastric cancer syndrome and invasive lobular carcinoma of the breast [8, 9].

More than 1000 studies have analyzed the role of E-Cadherin expression in cancer using immunhistochemistry. The data show that E-Cadherin expression occurs in a wide range of human tumors and that not only reduced [10–16] but also elevated E-Cadherin protein levels [17–19] can be associated with unfavorable tumor parameters. However, the accumulated data on the prevalence of E-Cadherin expression is controversial in the literature. For example, E-Cadherin positivity has been described in 25 to 100% of invasive breast carcinoma of no special type [20–22], 32 to 100% of oral squamous cell carcinomas [23–25], 31 to 100% of intestinal gastric carcinomas [26–28], 26 to 100% of colorectal adenocarcinomas [29–31], and 5 to 54% of clear cell renal cell carcinomas [17, 32, 33].

These conflicting data make it virtually impossible to compare different tumor types with respect to their E-Cadherin expression levels. Because highly variable results have been reported even from the same histological tumor subtype in different studies using different experimental conditions and scoring criteria, it appears likely that many of the controversial data in the literature are due likely due to the use of different antibodies, immunostaining protocols, and criteria to categorize E-Cadherin in these studies.

Knowledge on the relative expression levels of E-Cadherin in different tumor types would substantially add to the understanding of the role of this protein in these cancers. In addition, data on rare tumor types or subtypes are lacking. To better understand the prevalence and significance of E-Cadherin expression in across human cancers, a comprehensive study analyzing a large number of neoplastic and non-neoplastic tissues under highly standardized conditions is needed. Therefore, E-Cadherin expression was analyzed in more than 14,000 tumor tissue samples from 112 different tumor types and subtypes as well as 76 non-neoplastic tissue categories by immunohistochemistry (IHC) in a tissue microarray (TMA) format in this study.

## Methods

### Aim, design and setting of the study

This study aimed at the comprehensive analysis of E-Cadherin expression across all human types of normal tissue as well as more than 100 different human tumor types and subtypes. The tissue microarray format was employed to allow for a highly standardized analysis using immunohistochemistry with identical experimental conditions and identical amount of analyzed tissue for all > 14,000 tissue samples included in the study. The result is a ranking list of human tumor types according to the level of E-Cadherin expression.

### Tissue microarrays (TMAs)

Our normal tissue TMA was composed of 8 samples from 8 different donors for each of 76 different normal tissue types (608 samples on one slide). The cancer TMAs contained a total of 14,637 primary tumors from 112 tumor types and subtypes. Detailed histopathological data on grade, tumor stage (pT), and lymph node status (pN) status were available from 4478 cancers (breast, colorectal, and kidney carcinoma). Clinical follow up data were available from 1183 breast cancer and 1174 renal cell cancer (RCC) patients with a median follow-up time of 49 and 48 months, respectively (range 1–88/1–250). The composition of both normal and cancer TMAs is described in detail in the results section. All samples come from the archives of the Institute of Pathology, University Hospital of Hamburg, Germany, the Institute of Pathology, Clinical Center Osnabrueck, Germany, and Department of Pathology, Academic Hospital Fuerth, Germany. Tissues were fixed in 4% buffered formalin and then embedded in paraffin. TMA tissue spot diameter was 0.6 mm.

### Immunohistochemistry (IHC)

Freshly cut TMA sections were all immunostained on the same day and in a single run. Slides were deparaffinized with xylol, rehydrated through a graded alcohol series and exposed to heat-induced antigen retrieval for 5 min in an autoclave at 121 °C in pH 9 DakoTarget Retrieval Solution (Agilent; #S2367). Endogenous peroxidase activity was blocked with Dako peroxidase blocking solution (Agilent; #52023) for 10 min. Primary antibody specific for E-Cadherin (mouse monoclonal, MSVA-035, MS Validated Antibodies, Hamburg, Germany) was applied at 37 °C for 60 min at a dilution of 1:300. Bound antibody was then visualized using the EnVision Kit (Agilent, CA, USA; #K5007) according to the manufacturer’s directions. The sections were counterstained with haemalaun.

One trained pathologist evaluated all stainings. Normal tissue spots were scored as negative (no detectable staining) or positive (detectable staining of any intensity). For tumor tissue spots, the staining was scored semiquantitatively. Four staining categories were identified based on the staining intensity (0, 1+, 2+, 3+) of the tumor cells and the fraction of stained tumor cells in each tissue spot. These categories included “negative” (no detectable staining), “weak” (1+ staining intensity in ≤70% of tumor cells or 2+ intensity in ≤30% of tumor cells), “moderate” (1+ staining intensity in > 70% of tumor cells, or 2+ intensity in 31–70% of tumor cells, or 3+ intensity in ≤30% of tumor cells), and “strong” (2+ intensity in > 70% of tumor cells or 3+ intensity in > 30% of of tumor cells).

### Statistics

Statistical calculations were performed with JMP 14 software (SAS Institute Inc., NC, USA). Contingency tables and the chi^2^-test were performed to search for associations between E-Cadherin and tumor phenotype. Survival curves were calculated according to Kaplan-Meier. The Log-Rank test was applied to detect significant differences between groups. A *p* value of ≤0.05 was considered as statistically significant.

## Results

### Technical issues

A total of 10,851 (74.1%) of 14,637 tumor samples were interpretable in our TMA analysis. The remaining 3786 (25.9%) samples were not interpretable due to the lack of unequivocal tumor cells or loss of the tissue spot during the technical procedures. On the normal tissue TMA, a sufficient number of samples were always analyzable for each tissue type to determine its E-cadherin expression status.

### E-Cadherin in normal tissue

**Fig. 1:**
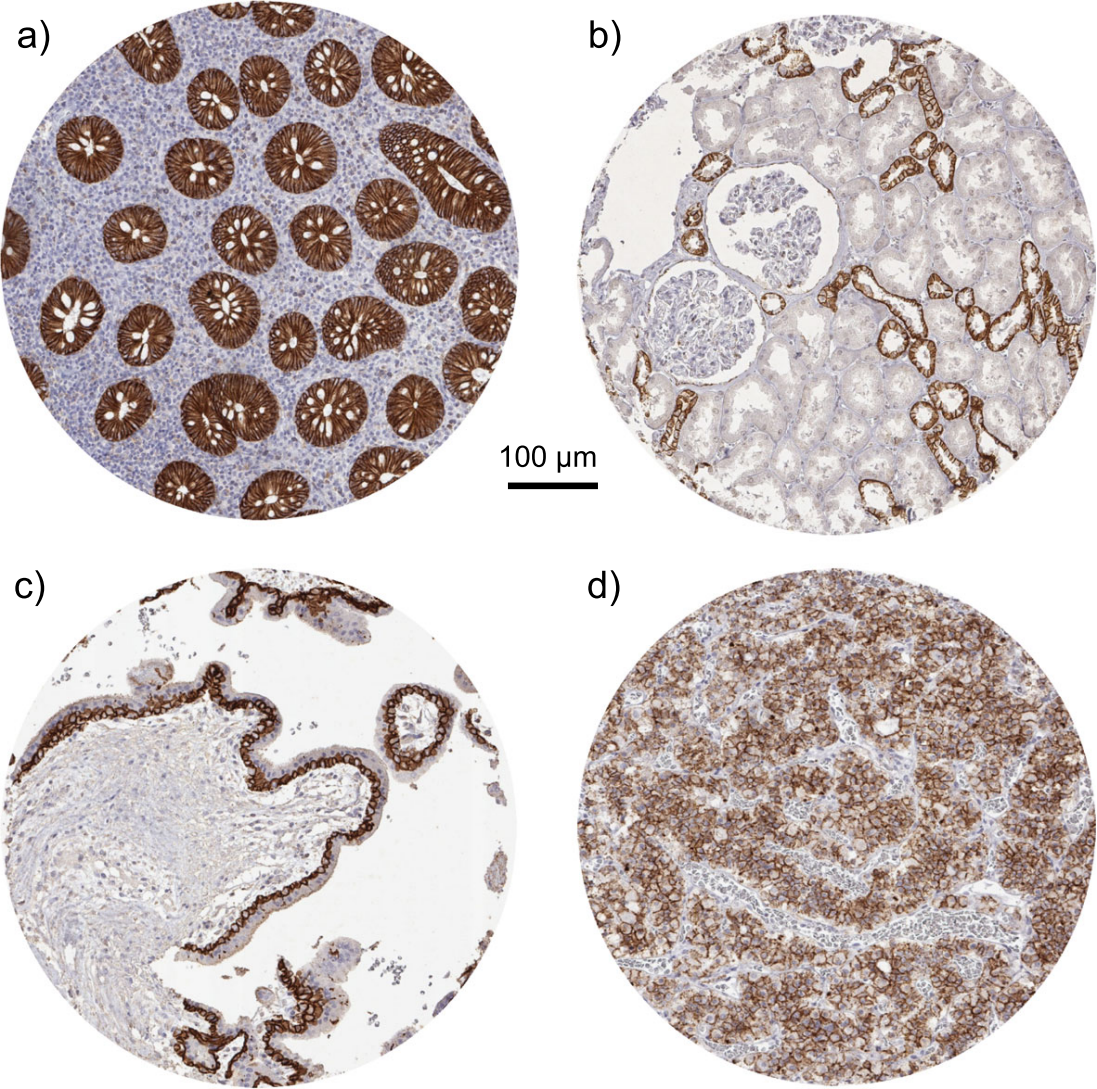
E-Cadherin expression in normal tissues. Moderate to strong E-Cadherin immunostaining is seen in epithelial cell of the appendix (**a**), in distal tubuli of the kidney (**b**), in the cytotrophoblastic layer of the placenta (**c**), in the pituitary gland (**d**)

A moderate to strong (2+/3+) membranous E-Cadherin staining was found in most epithelial cells of various organs (skin, lip, oral cavity, tonsils, salivary glands, esophagus, stomach, duodenum, ileum, appendix, colon, rectum, anal canal, gall bladder, liver, pancreas, ectocervix, endocervix, endometrium, fallopian tube, breast, thyroid gland, kidney pelvis, urinary bladder, prostate gland, seminal vesicle, epididymis, respiratory epithelium of bronchus and sinus paranasales, and lung (Fig. 1a). A distinct distribution of E-Cadherin expression were seen in the kidney, where only distal tubuli showed an E-Cadherin expression (Fig. 1b) and in the placenta, where only the cytotrophoblastic layer showed a positive staining (Fig. 1c). The anterior and posterior lobe of the pituitary gland showed a moderate to strong positive staining (Fig. 1d). Lymphatic tissue sometimes showed weak staining and some small vessel-like structures were also positive. In the thymus, positive staining was seen in Hassall’s corpuscles. E-Cadherin immunostaining was absent in endothelium and media of the aorta, the heart, striated muscle, tongue muscle, myometrium of the uterus, muscularis of the gastrointestinal tract, kidney pelvis and urinary bladder, corpus spongiosum of the penis, testis, ovarian stroma, corpus luteum of the ovary, adrenal gland, fat, cerebellum and cerebrum.

### E-Cadherin in neoplastic tissues

**Fig. 2:**
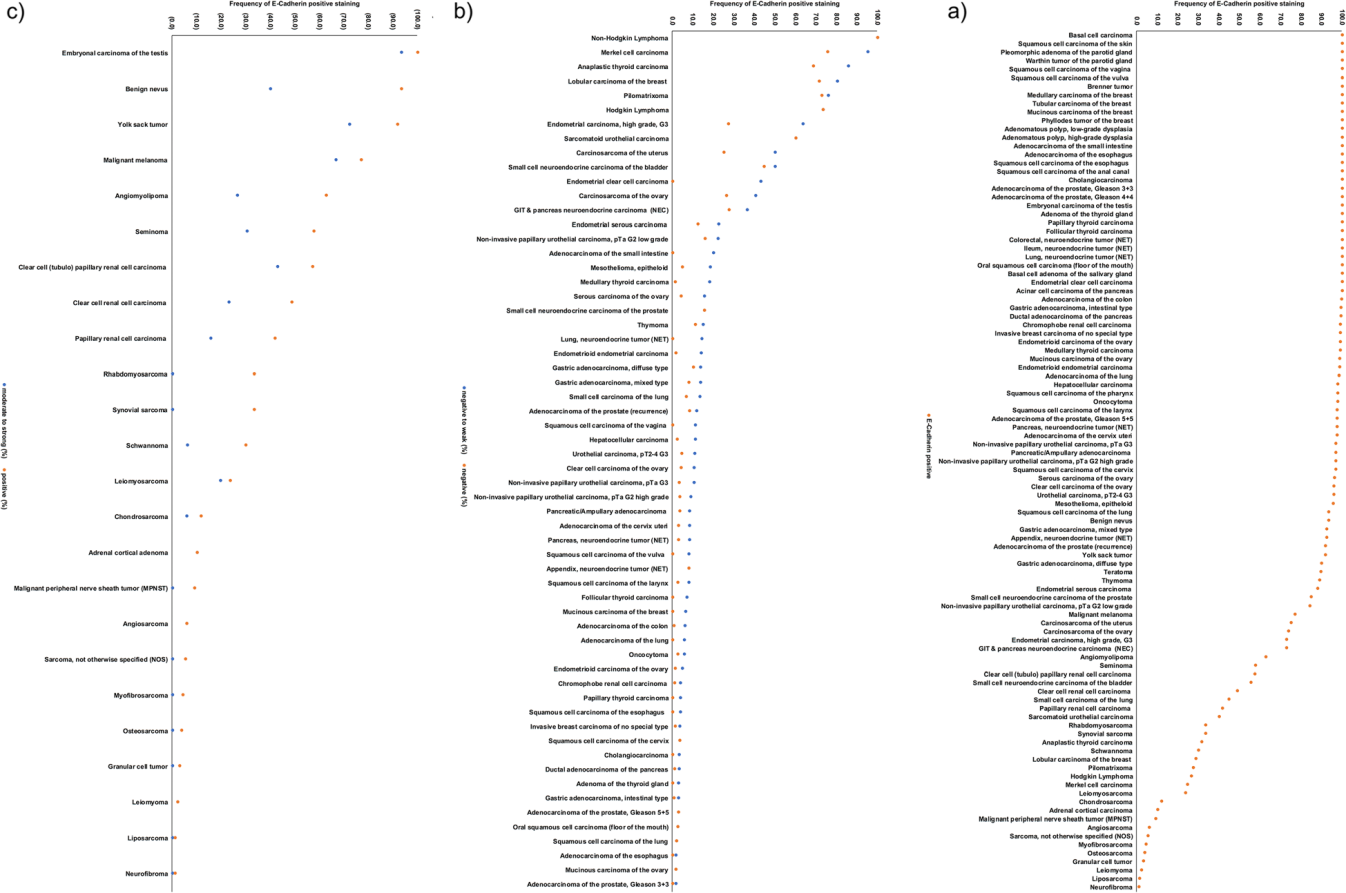
Ranking order of E-Cadherin immunostaining in tumors. **a** Tumor types and subtypes with positive (orange dots) staining (*n* = 101), **b** tumor types and subtypes from E-Cadherin positive normal cells with negative (orange dots) and negative to weak (blue dots) immunostaining (*n* = 60; not shown: 17 tumor types without E-Cadherin loss), and **c** tumor types and subtypes from E-Cadherin negative normal cells with positive (orange dots) and moderate to strong (blue dots) immunostaining (*n* = 24, not shown: 5 tumor types without E-Cadherin staining)

**Fig. 3:**
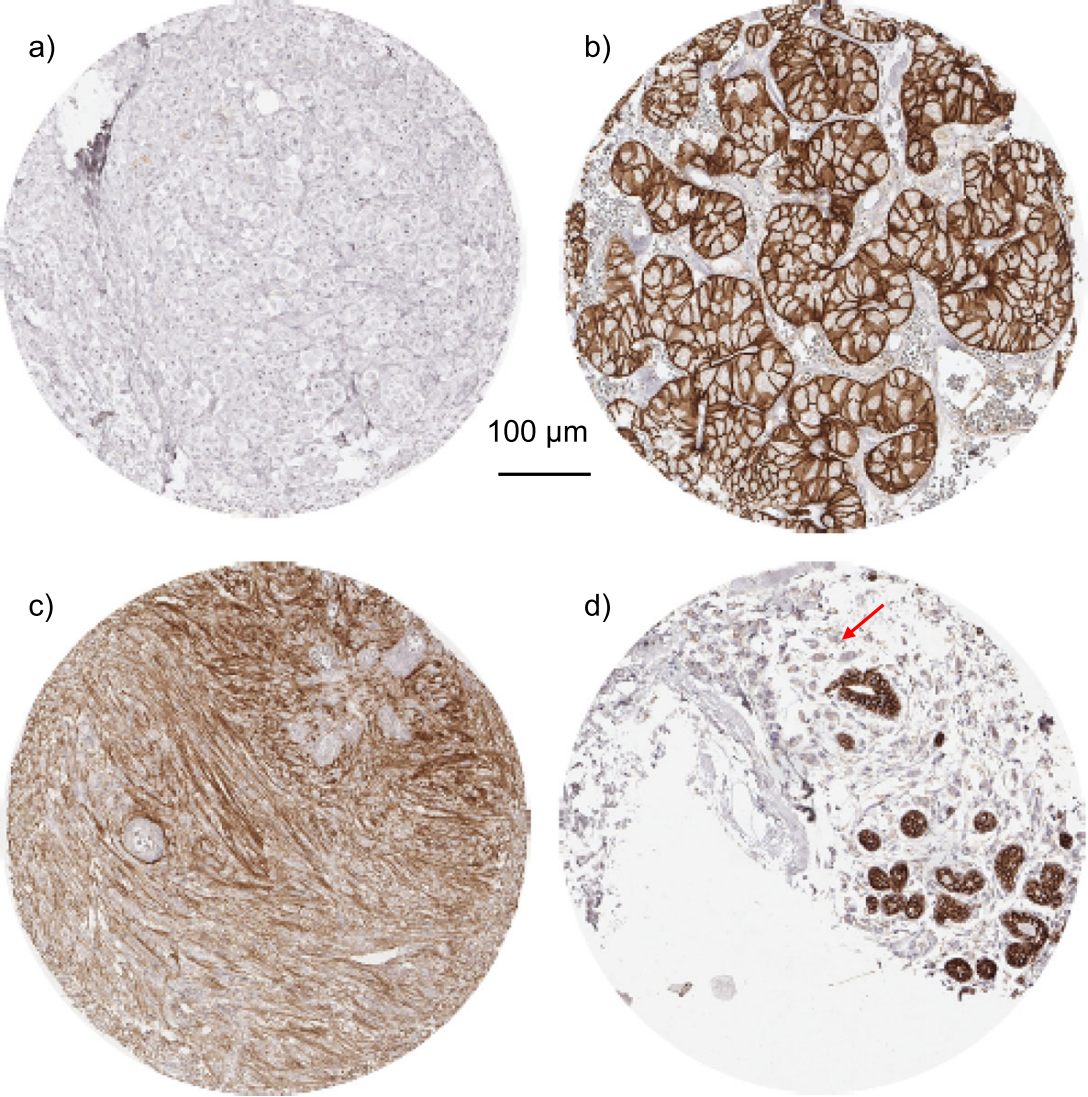
E-Cadherin expression in cancer tissue. **a** Absent E-Cadherin immunostaining in a castration resistent prostate cancer with discoherent growth pattern, **b** strong membranous E-Cadherin staining in a chromophobe renal cell carcinoma, **c** moderate intensity E-Cadherin staining in a Schwannoma, and **d** absence of E-Cadherin staining in the cells of an invasive lobular carcinoma (red arrow) in the vicinity of positively stained normal breast glands

Membranous E-Cadherin immunostaining was observed in 8819 (81.3%) of 10,851 interpretable tumors, including 7013 (64.6%) with strong, 927 (8.5%) with moderate, and 879 (8.1%) with weak staining. Overall, at least focal weak E-Cadherin immunostaining could be detected in 101 (90.2%) of 112 different tumor types and tumor subtypes (Table 1, Fig. 2a). In tumor entities derived from normally E-Cadherin positive cells types (Fig. 2b), at least a weak E-Cadherin positivity was preserved in 35 (45.5%) of 77 tumor entities in ≥99% of all interpretable examples and in 61 (79.2%) tumor entities in ≥90% of all analyzable cases. Tumors with the highest rates of E-Cadherin loss included Merkel cell carcinoma (75.6% negative), anaplastic thyroid carcinoma (68.6% negative), lobular carcinoma of the breast (71.5% negative), and sarcomatoid (60.0% negative) and small cell neuroendocrine carcinoma (44.4% negative) of the urinary bladder. In tumor entities derived from cell types normally negative for E-Cadherin (*n* = 29; Fig. 2c), a weak to strong E-Cadherin positivity could be detected in at least 10% of cases in 15 different tumor entities (51.7%). Tumors with the highest frequency of E-Cadherin upregulation included various subtypes of testicular germ cell tumors (positive 57 to 100%), melanocytic tumors (40 to 67%), and RCC (positive 42 to 57%). Representative images of E-Cadherin immunostaining in tumors are given in Fig. 3.

**Table 1:** E-Cadherin immunostaining in human tumors

Tumor type		n on TMA	E-Cadherin expression					
			analyzable (n)	negative (%)	weak (%)	moderate (%)	strong (%)	positive (%)
**Tumors of the skin**	Pilomatrixoma	35	33	72.7	3.0	15.2	9.1	27.3
	Basal cell carcinoma	48	33	0.0	0.0	3.0	97.0	100.0
	Benign nevus	29	15	6.7	53.3	33.3	6.7	93.3
	Squamous cell carcinoma of the skin	50	29	0.0	0.0	6.9	93.1	100.0
	Malignant melanoma	48	39	23.1	10.3	5.1	61.5	76.9
	Merkel cell carcinoma	46	41	75.6	19.5	2.4	2.4	24.4
**Tumors of the head and neck**	Squamous cell carcinoma of the larynx	50	39	2.6	5.1	0.0	92.3	97.4
	Squamous cell carcinoma of the pharynx	50	43	2.3	0.0	9.3	88.4	97.7
	Oral squamous cell carcinoma (floor of the mouth)	50	36	0.0	2.8	5.6	91.7	100.0
	Pleomorphic adenoma of the parotid gland	49	45	0.0	0.0	0.0	100.0	100.0
	Warthin tumor of the parotid gland	15	14	0.0	0.0	7.1	92.9	100.0
**Tumors of the lung, pleura and thymus**	Basal cell adenoma of the salivary gland	250	123	0.0	5.7	3.3	91.1	100.0
	Adenocarcinoma of the lung	127	54	1.9	0.0	9.3	88.9	98.1
	Squamous cell carcinoma of the lung	20	15	6.7	6.7	0.0	86.7	93.3
	Small cell carcinoma of the lung	76	56	55.4	3.6	16.1	25.0	44.6
	Mesothelioma, epitheloid	39	22	4.5	13.6	36.4	45.5	95.5
	Thymoma	29	27	11.1	3.7	14.8	70.4	88.9
**Tumors of the female genital tract**	Squamous cell carcinoma of the vagina	48	18	0.0	11.1	5.6	83.3	100.0
	Squamous cell carcinoma of the vulva	50	26	0.0	7.7	11.5	80.8	100.0
	Squamous cell carcinoma of the cervix	50	29	3.4	0.0	3.4	93.1	96.6
	Adenocarcinoma of the cervix uteri	50	37	2.7	5.4	5.4	86.5	97.3
	Endometrioid endometrial carcinoma	236	200	1.5	12.5	15.5	70.5	98.5
	Endometrial serous carcinoma	82	49	12.2	10.2	16.3	61.2	87.8
	Carcinosarcoma of the uterus	48	32	25.0	25.0	15.6	34.4	75.0
	Endometrial clear cell carcinoma	8	7	0.0	42.9	28.6	28.6	100.0
	Endometrial carcinoma, high grade, G3	13	11	27.3	36.4	0.0	36.4	72.7
	Endometrial stromal sarcoma	12	11	100.0	0.0	0.0	0.0	0.0
	Endometrioid carcinoma of the ovary	115	84	1.2	3.6	6.0	89.3	98.8
	Serous carcinoma of the ovary	567	480	4.0	11.5	21.3	63.3	96.0
	Mucinous carcinoma of the ovary	97	69	1.4	0.0	4.3	94.2	98.6
	Clear cell carcinoma of the ovary	54	48	4.2	6.3	10.4	79.2	95.8
	Carcinosarcoma of the ovary	47	42	26.2	14.3	11.9	47.6	73.8
	Brenner tumor	9	6	0.0	0.0	0.0	100.0	100.0
**Tumors of the breast**	Invasive breast carcinoma of no special type	1391	820	1.1	2.4	3.7	92.8	98.9
	Lobular carcinoma of the breast	294	158	71.5	8.9	6.3	13.3	28.5
	Medullary carcinoma of the breast	26	8	0.0	0.0	25.0	75.0	100.0
	Tubular carcinoma of the breast	27	6	0.0	0.0	0.0	100.0	100.0
	Mucinous carcinoma of the breast	58	16	0.0	6.3	6.3	87.5	100.0
	Phyllodes tumor of the breast	50	29	0.0	0.0	0.0	100.0	100.0
**Tumors of the digestive system**	Adenomatous polyp, low-grade dysplasia	50	39	0.0	0.0	0.0	100.0	100.0
	Adenomatous polyp, high-grade dysplasia	50	37	0.0	0.0	0.0	100.0	100.0
	Adenocarcinoma of the colon	1882	1644	0.5	5.5	9.7	84.3	99.5
	Adenocarcinoma of the small intestine	10	5	0.0	20.0	0.0	80.0	100.0
	Gastric adenocarcinoma, diffuse type	176	147	10.2	3.4	2.0	84.4	89.8
	Gastric adenocarcinoma, intestinal type	174	144	0.7	2.1	3.5	93.8	99.3
	Gastric adenocarcinoma, mixed type	62	52	7.7	5.8	1.9	84.6	92.3
	Adenocarcinoma of the esophagus	133	62	0.0	1.6	1.6	96.8	100.0
	Squamous cell carcinoma of the esophagus	124	55	0.0	3.6	0.0	96.4	100.0
	Squamous cell carcinoma of the anal canal	50	30	0.0	0.0	3.3	96.7	100.0
	Cholangiocarcinoma	114	93	0.0	3.2	7.5	89.2	100.0
	Hepatocellular carcinoma	50	45	2.2	8.9	11.1	77.8	97.8
	Ductal adenocarcinoma of the pancreas	612	437	0.9	2.3	9.6	87.2	99.1
	Pancreatic/Ampullary adenocarcinoma	89	61	3.3	4.9	3.3	88.5	96.7
	Acinar cell carcinoma of the pancreas	13	12	0.0	0.0	8.3	91.7	100.0
	Gastrointestinal stromal tumor (GIST)	50	40	100.0	0.0	0.0	0.0	0.0
**Tumors of the urinary system**	Non-invasive papillary urothelial carcinoma, pTa G2 low grade	177	95	15.8	6.3	11.6	66.3	84.2
	Non-invasive papillary urothelial carcinoma, pTa G2 high grade	141	91	3.3	5.5	6.6	84.6	96.7
	Non-invasive papillary urothelial carcinoma, pTa G3	187	125	3.2	7.2	8.8	80.8	96.8
	Urothelial carcinoma, pT2–4 G3	940	755	4.4	6.4	8.5	80.8	95.6
	Small cell neuroendocrine carcinoma of the bladder	18	18	44.4	5.6	11.1	38.9	55.6
	Sarcomatoid urothelial carcinoma	25	15	60.0	0.0	6.7	33.3	40.0
	Clear cell renal cell carcinoma	858	614	51.3	25.7	13.8	9.1	48.7
	Papillary renal cell carcinoma	255	180	58.3	26.1	6.1	9.4	41.7
	Chromophobe renal cell carcinoma	131	103	1.0	2.9	3.9	92.2	99.0
	Oncocytoma	177	127	2.4	3.1	12.6	81.9	97.6
	Clear cell (tubulo) papillary renal cell carcinoma	21	14	42.9	14.3	14.3	28.6	57.1
**Tumors of the male genital organs**	Adenocarcinoma of the prostate, Gleason 3 + 3	83	73	0.0	1.4	1.4	97.3	100.0
	Adenocarcinoma of the prostate, Gleason 4 + 4	80	70	0.0	0.0	0.0	100.0	100.0
	Adenocarcinoma of the prostate, Gleason 5 + 5	85	76	2.6	0.0	0.0	97.4	97.4
	Adenocarcinoma of the prostate (recurrence)	330	217	8.3	3.2	8.3	80.2	91.7
	Small cell neuroendocrine carcinoma of the prostate	17	13	15.4	0.0	0.0	84.6	84.6
	Seminoma	624	522	42.3	27.2	14.6	15.9	57.7
	Embryonal carcinoma of the testis	50	44	0.0	6.8	18.2	75.0	100.0
	Yolk sack tumor	50	36	8.3	19.4	5.6	66.7	91.7
	Teratoma	50	19	10.5	5.3	0.0	84.2	89.5
**Tumors of endocrine organs**	Adenoma of the thyroid gland	114	104	0.0	2.9	9.6	87.5	100.0
	Papillary thyroid carcinoma	392	354	0.0	3.7	7.6	88.7	100.0
	Follicular thyroid carcinoma	158	147	0.0	6.8	10.9	82.3	100.0
	Medullary thyroid carcinoma	107	84	1.2	16.7	39.3	42.9	98.8
	Anaplastic thyroid carcinoma	45	35	68.6	17.1	2.9	11.4	31.4
	Adrenal cortical adenoma	50	43	100.0	0.0	0.0	0.0	0.0
	Adrenal cortical carcinoma	26	20	90.0	0.0	0.0	10.0	10.0
	Phaeochromocytoma	50	42	100.0	0.0	0.0	0.0	0.0
	Appendix, neuroendocrine tumor (NET)	22	13	7.7	0.0	7.7	84.6	92.3
	Colorectal, neuroendocrine tumor (NET)	10	10	0.0	0.0	0.0	100.0	100.0
	Ileum, neuroendocrine tumor (NET)	49	43	0.0	0.0	0.0	100.0	100.0
	Lung, neuroendocrine tumor (NET)	19	14	0.0	14.3	7.1	78.6	100.0
	Pancreas, neuroendocrine tumor (NET)	102	75	2.7	5.3	4.0	88.0	97.3
	GIT & pancreas neuroendocrine carcinoma (NEC)	28	11	27.3	9.1	9.1	54.5	72.7
**Tumors of haemotopoetic and lymphoid tissues**	Hodgkin Lymphoma	45	38	73.7	0.0	5.3	21.1	26.3
	Non-Hodgkin Lymphoma	48	39	100.0	0.0	0.0	0.0	0.0
**Tumors of soft tissue and bone**	Tenosynovial giant cell tumor	45	37	100.0	0.0	0.0	0.0	0.0
	Granular cell tumor	53	34	97.1	2.9	0.0	0.0	2.9
	Leiomyoma	50	45	97.8	0.0	0.0	2.2	2.2
	Angiomyolipoma	91	64	37.5	35.9	9.4	17.2	62.5
	Angiosarcoma	73	51	94.1	0.0	0.0	5.9	5.9
	Dermatofibrosarcoma protuberans	21	12	100.0	0.0	0.0	0.0	0.0
	Ganglioneuroma	14	9	100.0	0.0	0.0	0.0	0.0
	Kaposi sarcoma	8	5	100.0	0.0	0.0	0.0	0.0
	Leiomyosarcoma	87	72	76.4	4.2	9.7	9.7	23.6
	Liposarcoma	132	89	98.9	1.1	0.0	0.0	1.1
	Malignant peripheral nerve sheath tumor (MPNST)	13	11	90.9	9.1	0.0	0.0	9.1
	Myofibrosarcoma	26	23	95.7	4.3	0.0	0.0	4.3
	Neurofibroma	117	99	99.0	1.0	0.0	0.0	1.0
	Sarcoma, not otherwise specified (NOS)	75	58	94.8	5.2	0.0	0.0	5.2
	Paraganglioma	41	38	100.0	0.0	0.0	0.0	0.0
	Primitive neuroectodermal tumor (PNET)	23	13	100.0	0.0	0.0	0.0	0.0
	Rhabdomyosarcoma	7	6	66.7	33.3	0.0	0.0	33.3
	Schwannoma	121	100	70.0	24.0	4.0	2.0	30.0
	Synovial sarcoma	12	9	66.7	33.3	0.0	0.0	33.3
	Osteosarcoma	43	27	96.3	3.7	0.0	0.0	3.7
	Chondrosarcoma	39	17	88.2	5.9	5.9	0.0	11.8

### E-Cadherin expression, tumor phenotype, and prognosis

**Fig. 4:**
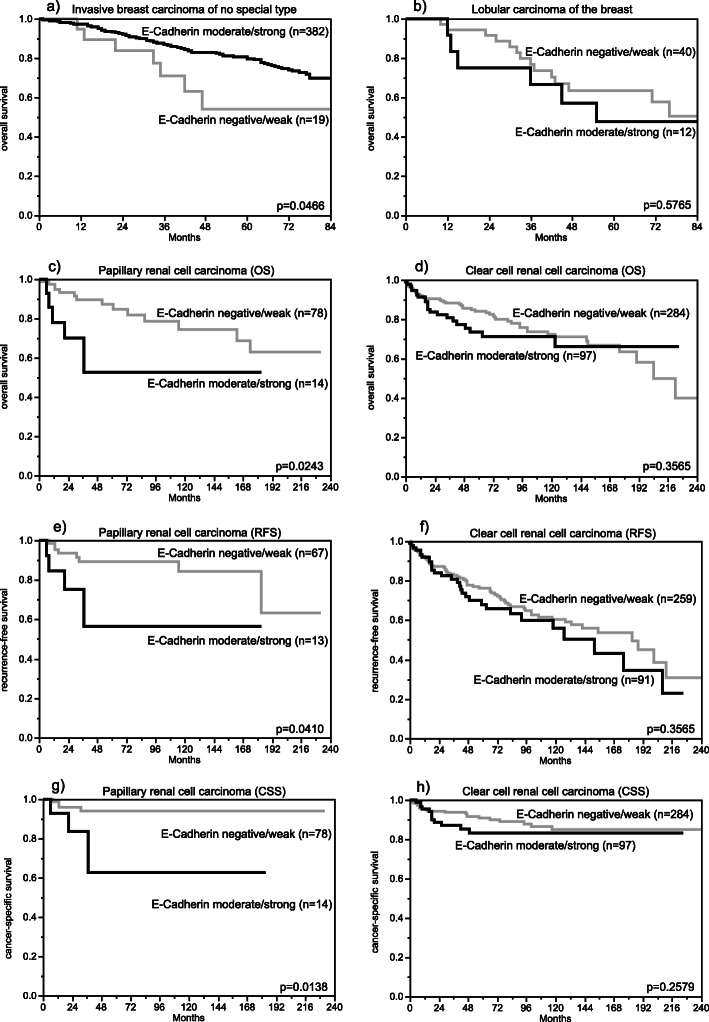
E-Cadherin immunostaining and patient prognosis. **a** in invasive breast carcinoma of no special type, **b** in lobular carcinoma of the breast, **c** papillary renal cell carcinoma, overall survival, (OS), **d** clear cell renal cell carcinoma (OS), **e** papillary renal cell carcinoma, recurrence-free survival (RFS), **f** clear cell renal cell carcinoma (RFS), **g** papillary renal cell carcinoma, cancer-specific survival (CSS), **h** clear cell renal cell carcinoma (CSS)

The relationship between E-Cadherin expression and clinico-pathological parameters or prognosis could be analyzed in three cancer types (breast, colorectal, and prostate cancer) derived from normally E-Cadherin positive cells and in two cancer types (papillary and clear cell RCC) derived from normally E-Cadherin negative cells. Reduced E-Cadherin expression was associated with high grade (*p* = 0.0009), triple negative receptor status (*p* = 0.0336), and reduced overall survival (*p* = 0.0466) in invasive breast carcinoma of no special type, triple negative receptor status (*p* = 0.0454) – but not with patient outcome - in lobular breast cancer, and with advanced pT stage (*p* = 0.0047) and nodal metastasis in colorectal cancer (*p* < 0.0001; Table 2, Fig. 4). In prostate cancer, E-Cadherin downregulation was more common in recurrent than in primary cancer. Negative to weak immunostaining was observed in 25 (11.5%) of 217 prostate cancer recurrences, and only in 3 (1.4%) of 219 primary prostate cancers (*p* < 0.0001). Increased E-Cadherin expression was related to advanced tumor stage (*p* = 0.0424), reduced overall survival (*p* = 0.0243), higher risk of recurrence (*p* = 0.0410) and cancer specific survival (*p* = 0.0138) in papillary RCC, and to advanced tumor stage (*p* = 0.0276) and high Thoenes grade (*p* = 0.0035) – but not patient prognosis – in clear cell RCC (Table 2, Fig. 4). E-Cadherin upregulation was also more commonly seen in malignant (43/331; 13.0%) than in benign (23/327; 7.0%) soft tissue tumors (*p* = 0.0104, Supplementary Figure 1).

**Table 2:** E-Cadherin immunostaining and tumor phenotype

			E-Cadherin immunostaining				
		n	negative (%)	weak (%)	moderate (%)	strong (%)	P
Invasive breast carcinoma of no special type	all cancers	757	1.1	2.6	3.6	92.7	
	pT1	392	1.3	2.6	2.8	93.4	0.6066
	pT2	276	0.7	2.9	3.6	92.8	
	pT3–4	60	1.7	3.3	8.3	86.7	
	Grade 1	111	0.0	0.0	0.0	100.0	0.0009
	Grade 2	393	1.8	3.6	3.6	91.1	
	Grade 3	252	0.4	2.4	5.2	92.1	
	pN0	360	1.1	1.9	3.3	93.6	0.5155
	pN ≥ 1	247	0.8	3.6	4.5	91.1	
	pM0	174	1.1	2.3	2.3	94.3	0.1374
	pM1	80	0.0	3.8	7.5	88.8	
	HER2 negative	563	1.1	2.5	4.4	92.0	0.2221
	HER2 positive	77	0.0	1.3	1.3	97.4	
	ER negative	133	1.5	3.8	6.8	88.0	0.1269
	ER positve	475	0.8	1.7	3.2	94.3	
	PR negative	250	1.2	2.0	5.2	91.6	0.7046
	PR positive	388	1.0	2.3	3.4	93.3	
	not Triple negative	499	0.8	1.6	3.2	94.4	0.0336
	Triple negative	93	2.2	4.3	8.6	84.9	
Lobular carcinoma of the breast	all cancers	93	65.6	9.7	9.7	15.1	
	pT1	40	72.5	10.0	5.0	12.5	0.7552
	pT2	35	62.9	8.6	11.4	17.1	
	pT3–4	16	56.3	6.3	18.8	18.8	
	Grade 1	4	50.0	25.0	0.0	25.0	0.3431
	Grade 2	76	67.1	10.5	10.5	11.8	
	Grade 3	13	61.5	0.0	7.7	30.8	
	pN0	54	64.8	9.3	11.1	14.8	0.5989
	pN ≥ 1	11	70.0	3.3	6.7	20.0	
	pM0	21	71.4	0.0	9.5	19.0	0.9970
	pM1	11	72.7	0.0	9.1	18.2	
	HER2 negative	65	69.2	7.7	12.3	10.8	0.8664
	HER2 positive	1	100.0	0.0	0.0	0.0	
	ER negative	8	62.5	0.0	37.5	0.0	0.0909
	ER positve	47	70.2	8.5	8.5	12.8	
	PR negative	30	73.3	0.0	16.7	10.0	0.0672
	PR positive	34	67.6	11.8	5.9	14.7	
	not Triple negative	47	70.2	8.5	8.5	12.8	0.0454
	Triple negative	5	40.0	0.0	60.0	0.0	
Colorectal cancers	all cancers	1570	0.5	5.5	9.7	84.3	
	pT1	63	0.0	6.3	4.8	88.9	0.0047
	pT2	313	0.0	4.8	5.4	89.8	
	pT3	859	0.7	5.2	9.8	84.3	
	pT4	321	0.6	6.9	14.3	78.2	
	pN0	808	0.4	4.7	5.9	89.0	< 0.0001
	pN+	732	0.7	6.4	13.8	79.1	
	V0	1135	0.3	5.2	7.8	86.8	< 0.0001
	V+	396	1.0	6.8	15.2	77.0	
	L0	595	0.3	5.2	5.5	88.9	< 0.0001
	L1	922	0.7	6.0	12.5	80.9	
	left	1142	0.4	6.0	9.5	84.1	0.4828
	right	422	0.7	4.3	10.2	84.8	
	microsatellite instable	80	1.3	10.0	7.5	81.3	0.1616
	microsatellite stable	1096	0.3	4.6	8.1	87.0	
	RAS mutation	328	0.9	7.3	8.5	83.2	0.1609
	RAS wild type	420	0.0	7.1	9.5	83.3	
	BRAF V600E mutation	18	0.0	16.7	11.1	72.2	0.3486
	BRAF wild type	122	0.8	4.9	8.2	86.1	
Clear cell renal cell carcinoma	all cancers	569	51.1	25.1	14.2	9.5	
	ISUP 1	179	62.0	20.7	11.7	5.6	< 0.0001
	ISUP 2	185	45.9	30.8	14.6	8.6	
	ISUP 3	163	42.3	27.6	16.6	13.5	
	ISUP 4	34	70.6	11.8	11.8	5.9	
	Fuhrmann 1	25	72.0	20.0	8.0	0.0	0.1532
	Fuhrmann 2	334	51.5	26.3	13.8	8.4	
	Fuhrmann 3	169	45.6	25.4	16.6	12.4	
	Fuhrmann 4	40	60.0	17.5	12.5	10.0	
	Thoenes 1	194	61.3	22.7	10.8	5.2	0.0036
	Thoenes 2	318	44.0	28.0	16.7	11.3	
	Thoenes 3	56	57.1	17.9	12.5	12.5	
	UICC 1	238	50.8	26.1	15.5	7.6	0.1201
	UICC 2	27	51.9	25.9	7.4	14.8	
	UICC 3	76	31.6	36.8	15.8	15.8	
	UICC 4	62	53.2	24.2	12.9	9.7	
	pT1	319	52.4	23.8	16.0	7.8	0.0276
	pT2	60	66.7	16.7	6.7	10.0	
	pT3–4	185	44.3	30.3	13.5	11.9	
	pN0	100	50.0	25.0	12.0	13.0	0.4847
	pN ≥ 1	14	28.6	35.7	14.3	21.4	
	pM0	86	44.2	32.6	14.0	9.3	0.5788
	pM ≥ 1	64	54.7	23.4	12.5	9.4	
Papillary renal cell carcinoma	all cancers	131	59.0	25.9	5.8	9.3	
	ISUP 1	26	69.2	23.1	3.8	3.8	0.0207
	ISUP 2	65	52.3	29.2	6.2	12.3	
	ISUP 3	46	63.0	23.9	4.3	8.7	
	ISUP 4	1	0.0	0.0	100.0	0.0	
	Fuhrmann 1	1	100.0	0.0	0.0	0.0	0.7761
	Fuhrmann 2	92	58.7	27.2	4.3	9.8	
	Fuhrmann 3	42	57.1	26.2	7.1	9.5	
	Fuhrmann 4	3	66.7	0.0	33.3	0.0	
	Thoenes 1	33	63.6	30.3	6.1	0.0	0.018
	Thoenes 2	96	56.3	27.1	4.2	12.5	
	Thoenes 3	9	66.7	0.0	22.2	11.1	
	UICC 1	77	54.5	31.2	5.2	9.1	0.0067
	UICC 2	10	100.0	0.0	0.0	0.0	
	UICC 3	3	33.3	33.3	33.3	0.0	
	UICC 4	9	55.6	0.0	11.1	33.3	
	pT1	98	56.1	31.6	4.1	8.2	0.0424
	pT2	26	76.9	11.5	3.8	7.7	
	pT3–4	10	40.0	10.0	20.0	30.0	
	pN0	14	50.0	42.9	0.0	7.1	0.0368
	pN ≥ 1	7	57.1	0.0	14.3	28.6	
	pM0	23	60.9	26.1	4.3	8.7	0.2792
	pM ≥ 1	5	60.0	0.0	20.0	20.0

## Discussion

**Fig. 5:**
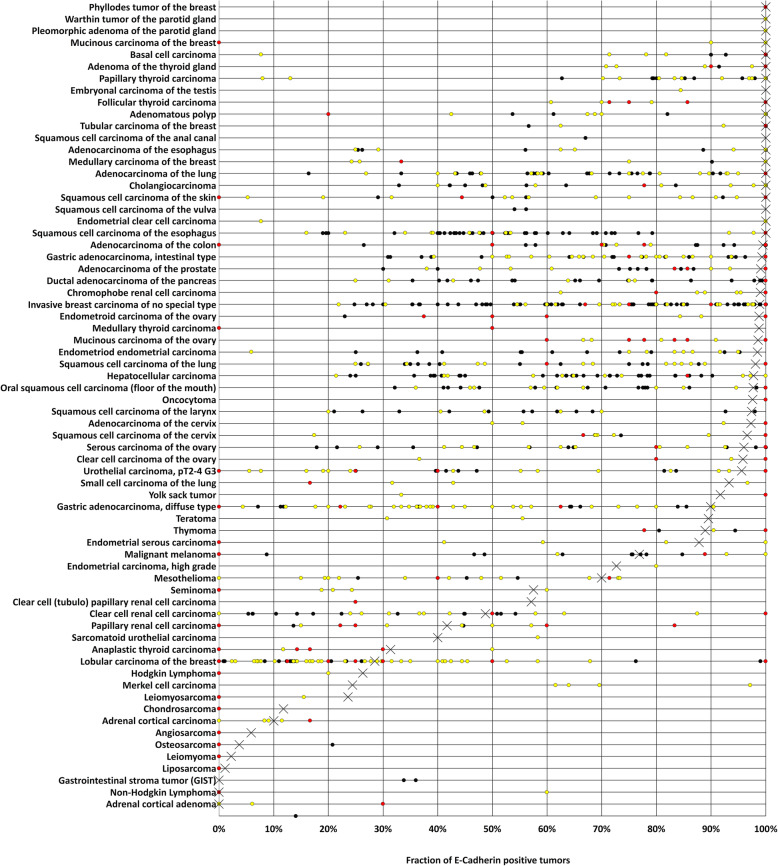
Graphical representation of E-Cadherin data from this study (x) in comparison with the previous literature. Red dots are used for studies involving 2–10 cases, yellow dots are used for studies involving 11–50 cases, and black dots are used for studies involving > 50 cases

More than 1000 studies have described E-Cadherin immunohistochemical expression in cancer. This abundance of data obtained by using varying staining protocols and criteria for interpretation have made it difficult to easily understand the relative importance of E-Cadherin expression in various cancer types. This standardized analysis of 10,851 cancers by IHC provides a comprehensive overview of E-Cadherin immunostaining in 112 different tumor types. The most significant result of our study is a rank order of cancers according to their frequency of E-Cadherin expression, which is shown in Fig. 5 together with earlier data from the literature. The finding that most tumor types show either very high or very low E-Cadherin expression frequencies reflects the fact that frequent and intense E-Cadherin immunostaining is commonly seen in cancers derived from E-Cadherin positive normal cell types while neo-expression of E-Cadherin is rare and usually low in neoplasia derived from E-Cadherin negative normal cells.

The group of cancers derived from E-cadherin positive normal cells and showing a particularly frequent loss of E-Cadherin expression included highly dedifferentiated cancers such as Merkel cell carcinoma, anaplastic thyroid cancer, dedifferentiated endometrium carcinoma, and sarcomatoid and small cell carcinomas of the urinary bladder. Together with other tumor types known to frequently show reduced E-Cadherin expression, such as invasive lobular breast cancer [34, 35], diffuse gastric carcinoma [28, 36], pseudopapillary neoplasm of the pancreas [37, 38] and plasmocytoid urothelial carcinoma [39, 40] these tumors morphologically have the loss of tumor cell cohesion in common. Given the pivotal role of E-Cadherin for cell-cell adhesion and maintenance of epithelial polarity [41, 42], it is tempting to speculate that noticeable effects of E-Cadherin downregulation on tumor morphology can appear. Molecular mechanisms for impaired E-Cadherin function include inactivating gene mutations, chromosomal deletions, and promotor hypermethylation which can occur in various combinations and can vary in frequency between cancer types [43–49]. Alternatively, the E-Cadherin function can be impaired by defects in other members of the E-Cadherin-Catenin complex – especially of alpha-catenin. Loss of alpha-catenin disrupts the structure of the E-Cadherin-Catenin complex, preventing the formation of cell-cell junctions between the actin cytoskeleton of adjacent cells (adherens junctions), thereby decreasing cell-cell adhesion [50–52].

The particularly high frequency of E-Cadherin loss in highly lethal cancers with dedifferentiated morphology already argues for a negative impact of E-Cadherin loss on the prognosis of cancer patients. Several aspects of our data support the concept that a reduced E-Cadherin expression may also be linked to unfavorable cancer features in tumors with less conspicuous morphology. In our study, reduced E-Cadherin expression was linked to high grade, triple negative receptor status and poor prognosis in invasive breast carcinoma of no special type, triple negative receptor status in lobular breast cancer, advanced pT stage and lymph node metastasis in colorectal cancer as well as prostate cancer progression. Significant associations of reduced E-Cadherin expression with poor outcome in invasive breast cancer [10–12, 53–57], triple receptor negativity in breast cancer [58], poor outcome and unfavorable tumor phenotype in colorectal cancer [29, 30, 59, 60], and adverse features in prostate cancer [13, 14] have been reported by various other investigators. However, several other studies have not found associations between reduced E-Cadherin expression and unfavorable patient prognosis or tumor phenotype in breast [20, 34, 54, 61–63], colorectal [31, 64] and prostate cancer [65]. Furthermore, previous studies in bladder and pancreatic cancer, tumors for which we did not find links to unfavorable tumor features, have provided inconsistent results, either suggesting [66–68] or rejecting [19, 69, 70] a prognostic role of reduced E-Cadherin expression. Overall, these data seem to suggest that reduced E-Cadherin expression is linked to unfavorable tumor outcome to some extent but cannot be considered a key indicator for aggressive disease course. This notion is also supported by the somewhat better prognosis of lobular breast cancer, a tumor with a particularly high rate of E-Cadherin loss, as compared to invasive breast cancer of no special type, a tumor which is mostly E-Cadherin positive [35, 71]. Also, the fact that two benign tumors - oncocytoma and non-invasive papillary urothelial pTaG2 low grade carcinoma - showed occasional E-Cadherin loss suggests that reduced or absent E-Cadherin immunostaining is not invariably linked to tumor malignancy. Given the high E-Cadherin expression in normal urothelium and the high positivity rate in invasive urothelial cancer, the comparatively high number of non-invasive papillary urothelial pTaG2 low grade carcinoma with loss of E-Cadherin immunostaining was highly unexpected. However, since large non-invasive papillary urothelial pTa tumors are often transported in containers with more tumor than formalin, it cannot be excluded, that these findings are caused by fixation artifacts [72, 73].

Upregulation of E-Cadherin as compared to normal cells was observed in 24 different tumor types in this study. The fact that the highest frequency of E-Cadherin positivity was seen in germ cell tumors may reflect the pluripotency of their precursor cells, which often results in a variable degree of epithelial differentiation in these tumors. The next large tumor categories with frequent E-Cadherin upregulation are papillary and clear cell RCCs derived from E-Cadherin negative proximal tubuli, melanocytic tumors, as well as several sarcoma types derived from E-Cadherin negative mesenchymal cells. Our data suggest that E-Cadherin upregulation is associated with increased cancer aggressiveness in these tumors. High E-Cadherin levels were more commonly seen in malignant than in benign soft tissue tumors, more frequent in leiomyosarcoma than in leiomyoma, associated with high grade in clear cell RCC and linked to poor prognosis in papillary RCC in this study. These observations are in line with one earlier study reporting unfavorable tumor properties in RCC with high E-Cadherin expression [17]. However, other authors could not confirm these results [74, 75]. What cellular function of E-Cadherin may be driving cancer progression in the case of protein upregulation is unclear. Two studies demonstrated, that E-Cadherin upregulation may lead to anoikis suppression, rapid formation of multicellular spheroids and allows therefore anchorage-independent cell growth in Ewing tumor cells [76] and oral squamous cell carcinoma cells [77]. It is also possible, that E-cadherin upregulation simply reflects aberrant differentiation or dedifferentiation of cancer cells and does not itself play a specific biological role.

## Conclusion

E-Cadherin is consistently expressed in the vast majority of epithelial cancers. Both loss of E-Cadherin expression in cancers derived from E-Cadherin positive normal cells and upregulation in malignancies derived from E-cadherin negative normal tend to be linked to unfavorable tumor phenotype and disease outcome. Diagnosis of lobular breast cancer and distinction of chromophobe from clear cell carcinoma remain the best diagnostic applications of E-Cadherin IHC.

## Supplementary Information

**Additional file 1 **: **Supplementary Figure 1**. Difference of E-Cadherin expression between benigne (including hemangiomas, ganglioneuromas, glomus tumors, granular cell tumors, myopericytomas, neurofibromas, paragangliomas and schwannomas) and maligne (including all types of sarcomas, dermatofibrosarcoma protuberans, mesotheliomas, and primitive neuroectodermal tumors) soft tissue tumors.

## Data Availability

The datasets used and/or analyzed during the current study are available from corresponding author on reasonable request.

## Abbreviations

IHC: Immunohistochemistry
p120-ctn: P120-catenin
pN: Pathological lymph node status
pT: Pathological tumor stage
RCC: Renal cell carcinoma
TMA: Tissue microarray
