# Supplementary material for: E-Cadherin expression in human tumors: a tissue microarray study on 10,851 tumors
Source: Biomark Res. 2021 Jun 5;9:44. doi: 10.1186/s40364-021-00299-4 (PMC8180156; doi:10.1186/s40364-021-00299-4)
Supplement: Supplementary file 1 — Additional file 1 : Supplementary Figure 1. Difference of E-Cadherin expression between benigne (including hemangiomas, ganglioneuromas, glomus tumors, granular cell tumors, myopericytomas, neurofibromas, paragangliomas and schwannomas) and maligne (including all types of sarcomas, dermatofibrosarcoma protuberans, mesotheliomas, and primitive neuroectodermal tumors) soft tissue tumors. [file 40364_2021_299_MOESM1_ESM.pdf]

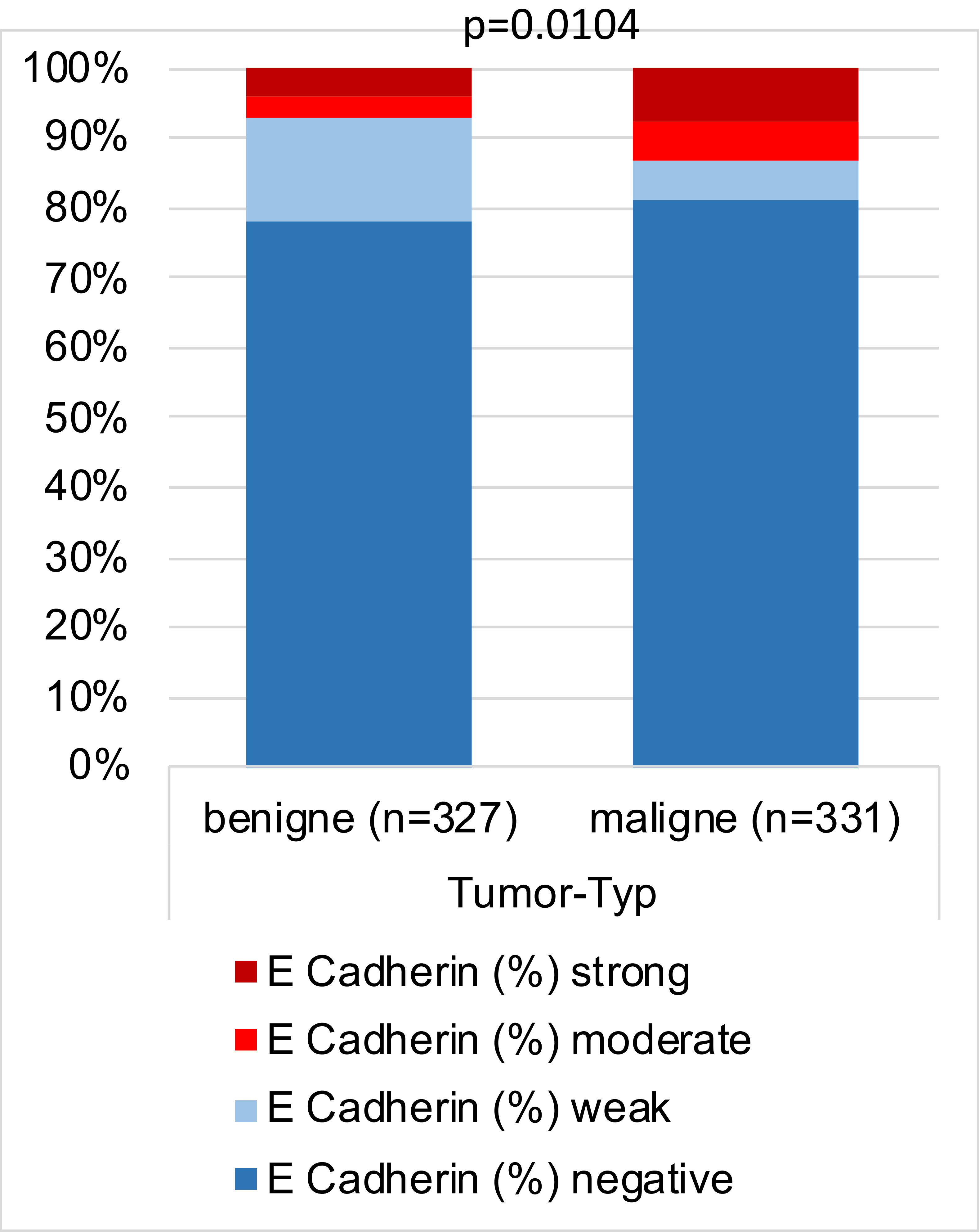

p=0.0104
100%
90%
80%
70%
60%
50%
40%
30%
20%
10%
0%
benigne (n=327)
maligne (n=331)
Tumor-Typ
E Cadherin (%) strong
E Cadherin (%) moderate
E Cadherin (%) weak
E Cadherin (%) negative
